# Increased blood-brain barrier hyperpermeability coincides with mast cell activation early under cuprizone administration

**DOI:** 10.1371/journal.pone.0234001

**Published:** 2020-06-08

**Authors:** John Shelestak, Naveen Singhal, Lana Frankle, Riely Tomor, Sarah Sternbach, Jennifer McDonough, Ernest Freeman, Robert Clements

**Affiliations:** 1 Department of Biological Sciences, School of Biomedical Sciences, Kent State University, Kent, Ohio, United States of America; 2 Department of Biochemistry, All India Institute of Medical Sciences, Rishikesh, Uttarakhand, India; Hungarian Academy of Sciences, HUNGARY

## Abstract

The cuprizone induced animal model of demyelination is characterized by demyelination in many regions of the brain with high levels of demyelination in the corpus callosum as well as changes in neuronal function by 4–6 weeks of exposure. The model is used as a tool to study demyelination and subsequent degeneration as well as therapeutic interventions on these effects. Historically, the cuprizone model has been shown to contain no alterations to blood-brain barrier integrity, a key feature in many diseases that affect the central nervous system. Cuprizone is generally administered for 4–6 weeks to obtain maximal demyelination and degeneration. However, emerging evidence has shown that the effects of cuprizone on the brain may occur earlier than measurable gross demyelination. This study sought to investigate changes to blood-brain barrier permeability early in cuprizone administration. Results showed an increase in blood-brain barrier permeability and changes in tight junction protein expression as early as 3 days after beginning cuprizone treatment. These changes preceded glial morphological activation and demyelination known to occur during cuprizone administration. Increases in mast cell presence and activity were measured alongside the increased permeability implicating mast cells as a potential source for the blood-brain barrier disruption. These results provide further evidence of blood-brain barrier alterations in the cuprizone model and a target of therapeutic intervention in the prevention of cuprizone-induced pathology. Understanding how mast cells become activated under cuprizone and if they contribute to blood-brain barrier alterations may give further insight into how and when the blood-brain barrier is affected in CNS diseases. In summary, cuprizone administration causes an increase in blood-brain barrier permeability and this permeability coincides with mast cell activation.

## Introduction

The cuprizone (bis-cyclohexanone oxaldihydrazone) model is a widely used model of demyelination and remyelination in the study of demyelinating and degenerative diseases in the central nervous system (CNS).[1] Cuprizone is a copper chelator which has been shown to affect mitochondria in hepatic cells of the liver and oligodendrocytes in the CNS.[2] The alteration of oligodendrocyte mitochondria leads to demyelination by apoptosis of the oligodendrocytes. This toxic, diffuse demyelination differs from other models of Multiple Sclerosis (MS) and demyelination that involve inflammatory processes to damage or destroy oligodendrocytes creating lesions in the CNS.[3] Cuprizone causes this mitochondrial toxicity by impairing activity of copper dependent cytochrome oxidase leading to decreased oxidative phosphorylation resulting in demyelination caused by oligodendrocyte dysfunction.[4] It is also known that oligodendrocytes display structural abnormalities manifested as enlarged mitochondria within demyelinated regions (most notably the corpus callosum).[5] Enzymatic changes have been shown to occur throughout the CNS, even in regions that do not display detectable pathological changes. These changes were observed not only in oligodendrocytes containing large mitochondria but also in neurons during cuprizone treatment.[6] Studies have also shown that cuprizone induced demyelination causes increased local oxidative stress, down regulates expression of mitochondria-encoded genes and changes intra-axonal mitochondrial density within affected neurons.[7] Cuprizone treatment also exhibits strong CNS glial activation that contributes to the pathology observed. It has also been shown that cuprizone induced oligodendrocyte death requires microglia/macrophage recruitment and inflammatory cytokine release,[8] and that this activation of microglia may depend on astrocytic cytokine release.[9] Following activation from astrocytes, microglia induce the aforementioned apoptosis and are also responsible for the clearing of the debris which manifests the demyelination seen under cuprizone administration.[10] The effects of cuprizone can be measured in different regions of the brain but are most predominant in the corpus callosum and less so in the cortex.[11] These changes are also temporally separated, permitting studies designed to observe or manipulate the dynamic changes that eventually result in a cascade of events including CNS glial activation, cell death and demyelination.

The blood brain barrier, (BBB), is a structure with properties unique to the CNS, which allows for strict control over the influx and efflux of nutrients, cells, and waste from the CNS.[12] The vasculature is characterized by tightly bound endothelial cells, held in place by tight junction proteins, that prevent extravasation and passive diffusion across the vasculature.[13] The basement membrane, BM, is an area of extra cellular matrix created by ECs, pericytes, and astrocytes. This membrane surrounds the ECs and serves as an additional barrier for the CNS while also regulating signaling process of the vasculature.[14] Glial cells such as astrocytes, pericytes, and microglia also serve in the development, maintenance, and function of the BBB. Astrocytes extend out processes to the vasculature with end-feet the encircle the vessels. These end-feet have polarized membranes on the vascular face that are responsible for water balance and cell signaling with the endothelial cells.[15] Signals from these end-feet have been implicated in the development of cohesive vessels by endothelial cells.[16] Pericytes have been shown to be necessary in the polarization of astrocytic end-feet as well as endothelial tight-junction expression.[17] Microglia, CNS macrophages, contribute to angiogenesis by promoting endothelial cell fusion [18] and protect BBB integrity via release of TGFβ1 release.[19] Breakdown of the BBB is a significant factor in the progression of many neurodegenerative diseases and may be caused by many of these cell populations.[20] In MS, inflammation in the brain leads to damage to the blood-brain barrier, causing it to become leaky.[21] Breakdown of the BM by matrix metalloproteinases, (MMPs), allows for the infiltration of peripheral leukocytes in to the CNS.[22] While early investigations reported no alterations in BBB integrity during cuprizone administration,[23] evidence is emerging that BBB disruption precedes the demyelination and glial activation observed in cuprizone treatment.[24]

Evidence has been mixed on the effect the cuprizone model has on the maintenance of an intact blood-brain barrier throughout the length of the demyelination.[25,26] However, the presence of peripheral cells such as macrophages have been found in cuprizone treated mouse brains which seems to indicate that the blood brain barrier does not remain intact.[27] The early activity of certain MMPs during cuprizone administration indicates BBB disintegration prior to the activation and gliosis of astrocytes and microglia.[28] Mast cells are early-responding granulocytes that are associated with BBB changes in many different neurodegenerative diseases, ischemia, and many auto-immune reactions.[29,30] Mast cells have been shown to release MMPs in response to inflammation which cause BM breakdown and BBB disintegration.[31] In addition, the release of the mast cell granule content histamine has been shown to increase BBB permeability of horseradish peroxidase.[32] As such, the focus of the current study is to further evaluate the early effects of cuprizone treatment on BBB integrity, glial activation and mast cell responses. *In vivo* evaluation of demyelination, BBB permeability, tight junction and basement membrane protein expression, as well as glial and mast cell activation were evaluated under cuprizone treatment. Early mast cell activation coincided with the highest levels of BBB permeability and tight junction changes and predated CNS activation of astrocytes and microglia. These findings indicate that further evaluation of mast cells in BBB alterations during cuprizone administration needs to be undertaken to evaluate their potential as a therapeutic target.

## Materials and methods

### Ethics statement

All animal studies and procedures were approved by the Institutional Animal Care and Use Committee at Kent State University

### Animals and diet

C7BL/6J 7-week-old male mice were fed a special diet with 0.3% cuprizone mixed into chow (Harlan Teklad, Madison WI) *ad libitum* for 3 days, 1 week, or 2 weeks (*n* = 6). The cuprizone chow was kept at 2°C to prevent inactivation due to heat sensitivity.[33] The chow was replaced in the cages every other day to ensure only active cuprizone was being eaten by the mice. The mice were single housed in wire bottomed cages to prevent coprophagy on feces that may contain digested cuprizone. The cages were covered with filtered tops to prevent the powdered cuprizone from becoming airborne. Weight was measured every day throughout the 3 day, 1, and 2-week studies.

### Evans blue injection

Evans blue staining was performed to evaluate BBB permeability.[34] Control and cuprizone treated mice (*n* = 6) from 3 days, 1, 2-week experiments were injected with Evans Blue (Sigma Aldritch, St. Louis MO) 24 hours prior to sacrifice. The mice were injected intraperitoneally with a 2% w/v solution of Evans Blue in normal saline at 4mL/kg of mouse body weight.[35] The stain was left to circulate for 24 hours before any final MR imaging and sacrifice.

### Magnetic resonance imaging

Magnetic resonance imaging was performed on the mice prior to the initiation of cuprizone administration and after 2 weeks immediately preceding sacrifice. The mice were intraperitoneally injected with a mixture of 5 mg/kg butorphanol (Zoetis, Kalamazoo MI) and .04 mg/kg atropine (Vedco, St. Joseph, MO) 30 minutes before imaging and anesthesia was maintained via 1.75% vaporized isoflurane in O2 throughout imaging. Anesthesia was confirmed via toe pinch and respiration rate was monitored to ensure complete anesthesia using PC-Sam software (SA Instruments Inc, Stony Brook, NY). Scout images were taken to ensure proper orientation and positioning of the mouse before scan initiation. T1-weighted fast low angle shot (FLASH), T2-weighted rapid acquisition with relaxation enhancement (RARE), and diffusion weighted scans were performed on a Bruker ICON 1 T solid-state MRI (Bruker, MA) for each mouse with total scan times lasting an hour. After imaging, the mice were left to recover from anesthesia in a heated cage for 30 minutes before being returned to their cages in the animal facility. After the final imaging session for each experiment, mice were immediately sacrificed via rapid-cervical dislocation and the brains removed. MRI scans were analyzed with ImageJ for signal intensity changes in the corpus callosum. Acquisition parameters for T1: *T_R_* = 10 ms, *T_E_* = 746.440, matrix size 128 x 128 mm, slice thickness = 0.750 mm, FoV = 20 x 20 mm. T2: *T_R_* = 85 ms, *T_E_* = 2800.218, matrix size 128 x 128 mm, slice thickness = 0.750 mm, FoV = 20 x 20 mm. DW: *T_R_* = 32 ms, *T_E_* = 1500, matrix size 64 x 96 mm, slice thickness = 0.750 mm, FoV = 20 x 20 mm.

### Tissue processing

After removal, brains were halved and either immediately frozen at -80°C for western blot analysis or fixed in 4% paraformaldehyde (PFA) for staining. The tissues were fixed in 4% PFA for 24 hours before being washed in phosphate-buffered saline (PBS) for 24 hours. They were then sectioned using an EMS OTS-5000 PBS media cooled vibratome at a thickness of 200 microns, stored in multi-well plates and stained.

### Toluidine blue staining

Slides were stained in a 0.05% toluidine blue solution (Sigma Aldritch, St. Louis MO). A 1% working stock solution in 70% ethanol was dissolved in a 1% NaCl solution (pH 2.0~2.5) before each staining session. The slides were rehydrated in distilled water for 2 min before being submerged in the toluidine blue working solution for 3 min. They were then washed in distilled water for 2 min before being hydrated in consecutively higher concentrations of ethanol. The slides were then submerged in 3 soaks of xylene for 2 min to clear the tissue for imaging. Utilizing the metachromatic nature of toluidine blue, the stained tissue was fluoresced using a 488 nm laser [36] in order to image the mast cells which contained an altered Stokes shift in the metachromatically shifted dye.[37] This allowed imaging of the mast cells which were fluorescent under 488 nm illumination and the emission was captured at 647nm.[38] Images were obtained using an Olympus laser scanning confocal microscope and associated Fluoview software (Olympus America, Melville, NY) and analyzed using ImageJ.

### Immunohistochemistry

Free-floating, PFA fixed slices (200 μm) were blocked in 3% normal donkey serum, and permeabilized in PBS containing 0.5% Triton-X for 24 hours on a rotating shaker at room temperature with appropriate unlabeled primary antibodies. After 24 hours, the slices were washed 3x15 min in PBS. The slices were then stained with any labelled primaries or secondaries as needed for an additional 24 hours. The sections were washed again 3x15 min each and left in a final PBS wash overnight. Stained sections were then air dried on charged slides and cover slipped with Vectashield antifading mounting media (Vector Laboratories, Burlingame, CA).

Labelled primary antibodies used in the study include Glial Fibrillary Acidic Protein (GFAP) (1:1000) (Sigma Aldritch, St. Louis MO), DAPI (1:2000) (Invitrogen, Carlsbad, California) (1:2000), Fluoromyelin Green (Invitrogen, Carlsbad, California) (1:1000). Anti-IBA 1 (Wako Richmond VA., 1:250) was stained with the labeled secondary anti-rabbit AlexaFluor 647 (1:250) (Invitrogen, Carlsbad, California). Imaging was performed with an Olympus laser scanning confocal microscope and associated Fluoview software (Olympus America, Melville, NY) equipped with 5 laser lines. Data was analyzed using ImageJ and in-house software for visualization, cell counts, and intensity measures. Cell counts and morphological analysis were performed by a blind observer.

### Western blot analysis

After sacrifice, one hemisphere of the brain was separated during removal and immediately frozen at -80°C. The cortex and corpus callosum were separated out and 10% homogenates were made in RIPA lysis buffer with a glass homogenizer at 4°C for each of the 2 regions. Whole cell lysates from both brain regions were obtained and centrifuged at 8000x gravity for 10 min 4°C. The supernatants from each sample were collected and further centrifuged at 4°C. Protein concentration (mg/ml) was determined using Bradford Assay reagent (Bio-Rad). Proteins were then denatured in Laemilli buffer (Bio-Rad) over dry hot bath. The samples were electrophoresed in either 7.5% or 10% SDS-polyacrylamide gels and transferred to PVDF membranes for antibody staining. The membranes were blocked with BSA and incubated with primary antibodies overnight at 4°C, washed in tris-buffered saline with Tween 20 (TBST) and secondary antibodies conjugated with HRP. Blots were then developed using luminol (Santa Cruz Biotechnologies) and imaged using a GE ImageQuant LAS 4000 mini system. (Millipore Billerica, MA 1:500). Primary antibodies used were ZO-1 (1:500) (Invitrogen, Carlsbad, California), Claudin-5 (1:500) (Invitrogen, Carlsbad, California), Occludin (1:500) (Invitrogen, Carlsbad, California), MMP-9 (1:500) (Abcam, Cambridge, UK), Mast Cell Tryptase (1:500) (Abcam, Cambridge, UK), Collagen IV (1:500) (Millipore, Burlington, MA), GAPDH (1:1000) (Millipore, Burlington, MA) and Actin (1:50,000) (Millipore, Burlington, MA).

### Statistical analysis

Statistical analysis was performed using IBM SPSS Statistics 25 software with results presented as a mean ±standard error of mean. (SEM). Student’s t tests and repeated measures ANOVAs were run where appropriate. Statistical effect size was measured using Cohen’s d; small effect < 0.2, moderate effect < 0.5 and large effect size > 0.8. Only *P* values < 0.05 were considered statistically significant.

## Results

### Blood-brain barrier alterations occur 3 days after cuprizone administration

**Fig 1:**
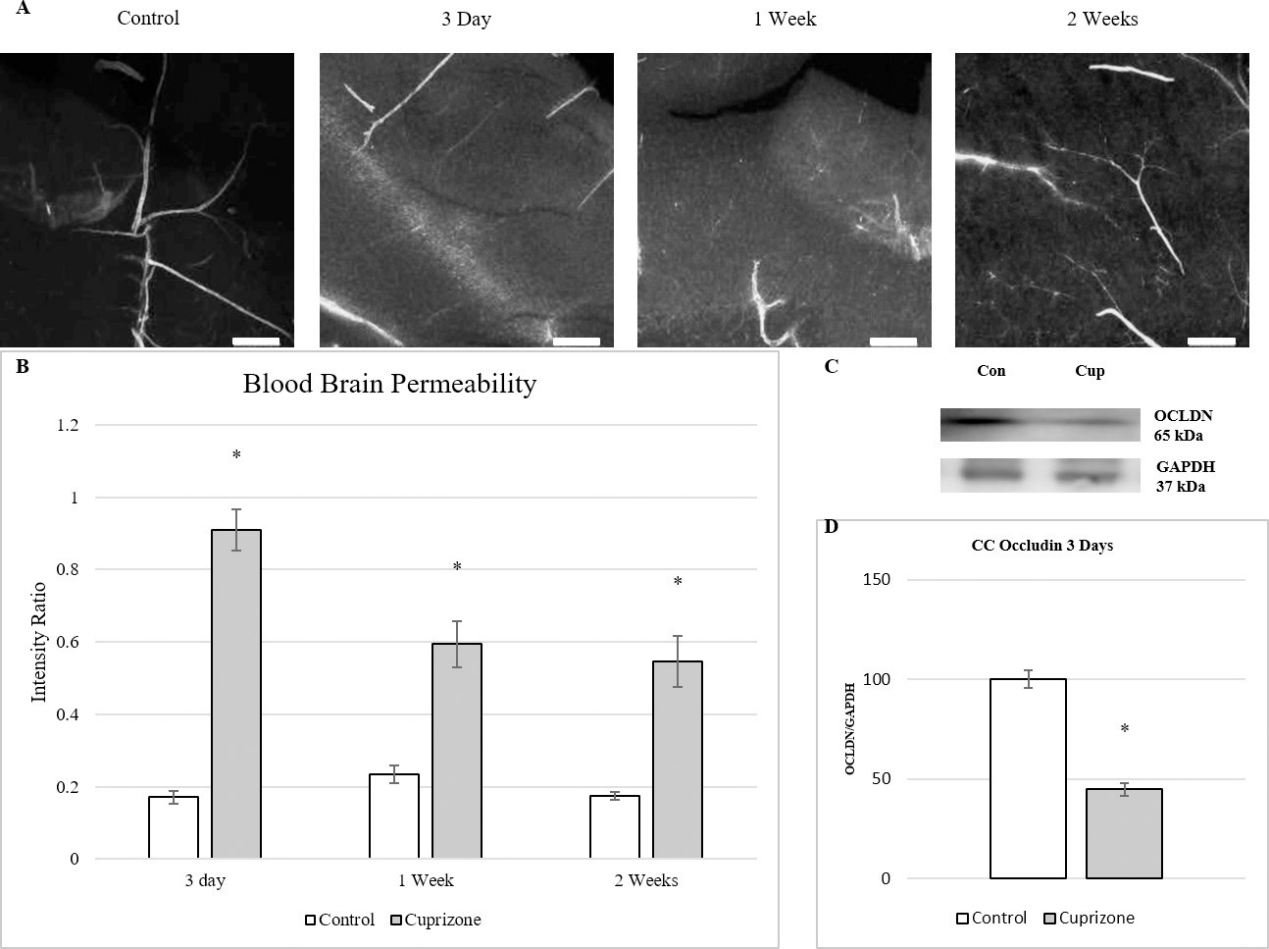
BBB impairment seen as early as 3 days into cuprizone administration. (A) Fluorescent images of vessels stained with Evans blue showing increased extravasation of the dye during the first 2 weeks of cuprizone treatment. 200x mag, Scale bar = 200 μm. (B) Intensity measures showed highest levels in vessel permeability at 3 days with significant differences measured at all time points (mean ± s.e.m.). *n* = 6, Asterisk denotes significance (*p* = < 0.05) (C) Western Blot analysis of corpus callosum. Occludin at 3 days of cuprizone administration. Representative blots of Occludin and GAPDH in mice after 3 days of cuprizone administration. (D) Densitometry of western blot shows significant decrease in Occludin levels compared to controls. Data are expressed as a percentage of control with the highest control set at 100. *n* = 3. Asterisk denotes significance (*p* = < 0.05).

Mice were injected intraperitoneally with Evans blue 24 hours prior to sacrifice. The brains were removed and fixed for fluorescent confocal imaging. Vascular permeability was evaluated using a modified version of fluorescent intensity measurements of tissue surrounding stained vessels from Evans Blue injected mice as reported by Schuh-Hofer et al.**(Fig 1A)**.[35] In short, vessels were thresholded and an ROI was made and measured for intensity and an ROI of approximately equal size, roughly 400–500μm was made in the area adjacent to the vessel and the ROI was measured for intensity. The intensity of the ROI of the adjacent tissue was normalized over the intensity of the corresponding vessel to correct for light scatter of bleed from hyper-intense areas. Imaged vessels showed diffuse staining of Evans blue in the tissue surrounding the vessels throughout the two weeks of cuprizone administration. Control vessels were well defined with little to no fluorescent signal in the surrounding tissue. Tissue treated with cuprizone showed much greater fluorescent intensity in the perivascular tissue with the highest levels occurring at 3 days and decreasing but still visible fluorescent signal through 2 weeks of cuprizone administration. The intensity of the Evans blue stained tissue was quantified and normalized by the signal of the associated vessel to establish the measure of vascular permeability **(Fig 1B)**.[35,39,40] Vascular permeability was measured at its highest ratio at 3 days after beginning cuprizone treatment, an over five-fold increase in intensity ratio, but remained significantly higher than controls across the duration of cuprizone treatment.

To better understand the changes in the BBB that lead to the increases in permeability, tight junction proteins was examined from the timepoints that exhibited the highest levels of permeability. Cortical and corpus callosum tissues were separated to examine if any regional differences existed and run through western analysis. Protein expression was analyzed at 3 days and 1 week to measure physiological changes that coincide with the highest levels of BBB permeability as measured above. Occludin, a transmembranal tight junctional protein, was found to be significantly reduced after 3 days of cuprizone administration by over 50% in tissue isolated from the corpus callosum **(Fig 1C and 1D).** Decreases in cortical occludin protein were seen but not measured as significant. The decreases in both corpus callosum and cortical tissues were recovered by 1 week. Zonula occludens-1 and claudin-5 were also measured and found to be lowered from control tissue but neither measured as statistically significant. Extracellular matrix proteins Collagen IV and matrix metalloproteinase 9 (MMP-9) were also analyzed to further examine BBB alterations via extracellular matrix modulation but none were found to be significantly altered in their expression.

### Blood-brain barrier changes precede measurable demyelination

**Fig 2:**
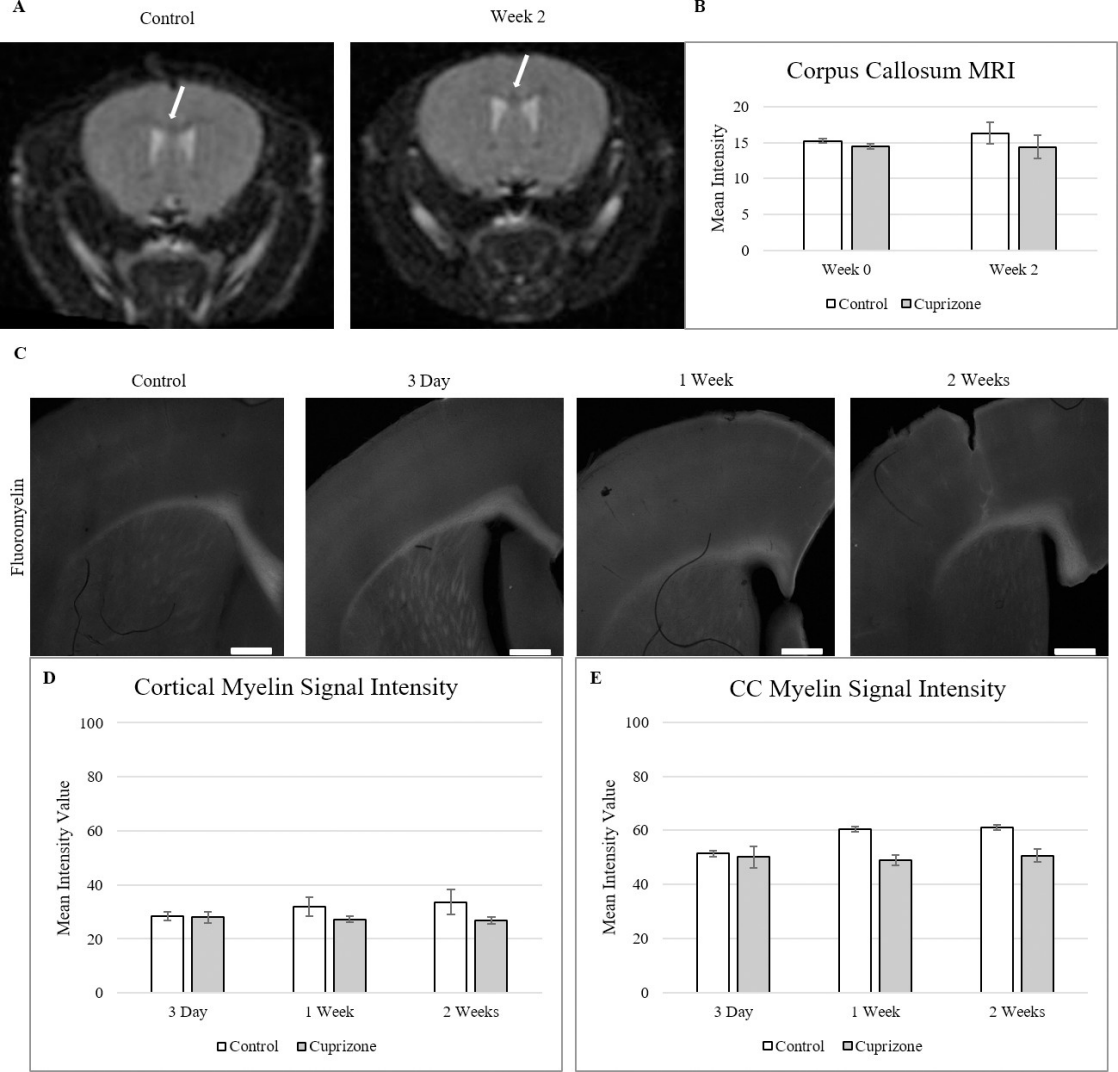
Myelin signal measures through 2 weeks of cuprizone treatment. **(**A) T2-RARE MR imaging of cuprizone induced demyelination in the corpus callosum. No visible changes in myelin signal in the corpus callosum was detected after 2 weeks of treatment and these did not differ between rostral and caudal regions as has been previously reported.[41,42] (B) Quantification of corpus callosum signal intensity shows no increases in T2 signal between groups or across time (mean± s.e.m.). Acquisition parameters: *T_R_* = 85 ms, *T_E_* = 2800.218 ms. White arrows indicate corpus callosum position. (*n = 6*, p < 0.05) (C) Fluoromyelin fluorescent staining of cortical and corpus callosum tissue in 3 days to 6 weeks of cuprizone treated tissue. (D-E) Quantification of cortex and corpus callosum showed no significant decrease in fluorescent signal through 2 weeks of treatment with cuprizone (mean± s.e.m.). Scale bar = 500 μm, *n* = 6. Asterisk denotes significance (*p* = < 0.05).

To better understand the role of the BBB alterations found in cuprizone pathology, demyelination was examined through the first 2 weeks of cuprizone administration. Demyelination in mice under cuprizone treatment was tracked via corpus callosum intensity under T2 MRI prior to the initiation of cuprizone treatment and at the end of the 2 weeks of treatment **(Fig 2A)**. A region of interest around the corpus callosum was created in ImageJ and mean pixel intensity was measured on each image. Under a T2-RARE imaging protocol, no visible changes appear in corpus callosum intensity between the control condition and 2 weeks. Repeated measures ANOVA showed also measured no significant differences between groups or across time in corpus callosum intensity measured under the T2 imaging (F = 19.056, p < 0.05) **(Fig 2B)**.

To corroborate the MR findings, demyelination was also measured in the cortex and corpus callosum via histological Fluoromyelin staining at 3 days, 1 and 2 weeks of cuprizone treatment **(Fig 2C)**. Fluoromyelin is a water-soluble dye that selectively binds myelin which was used to examine demyelination in both the cortex and corpus callosum.[43] Independent t-tests for both cortex and corpus callosum showed no changes in myelin fluorescent signal through the 2 weeks of cuprizone administration, matching the MRI data **(Fig 2D and 2E)**. This indicates that the hyperpermeability found during cuprizone administration predates measurable demyelination in within the CNS.

### CNS gliosis occurs after BBB hyperpermeability

**Fig 3:**
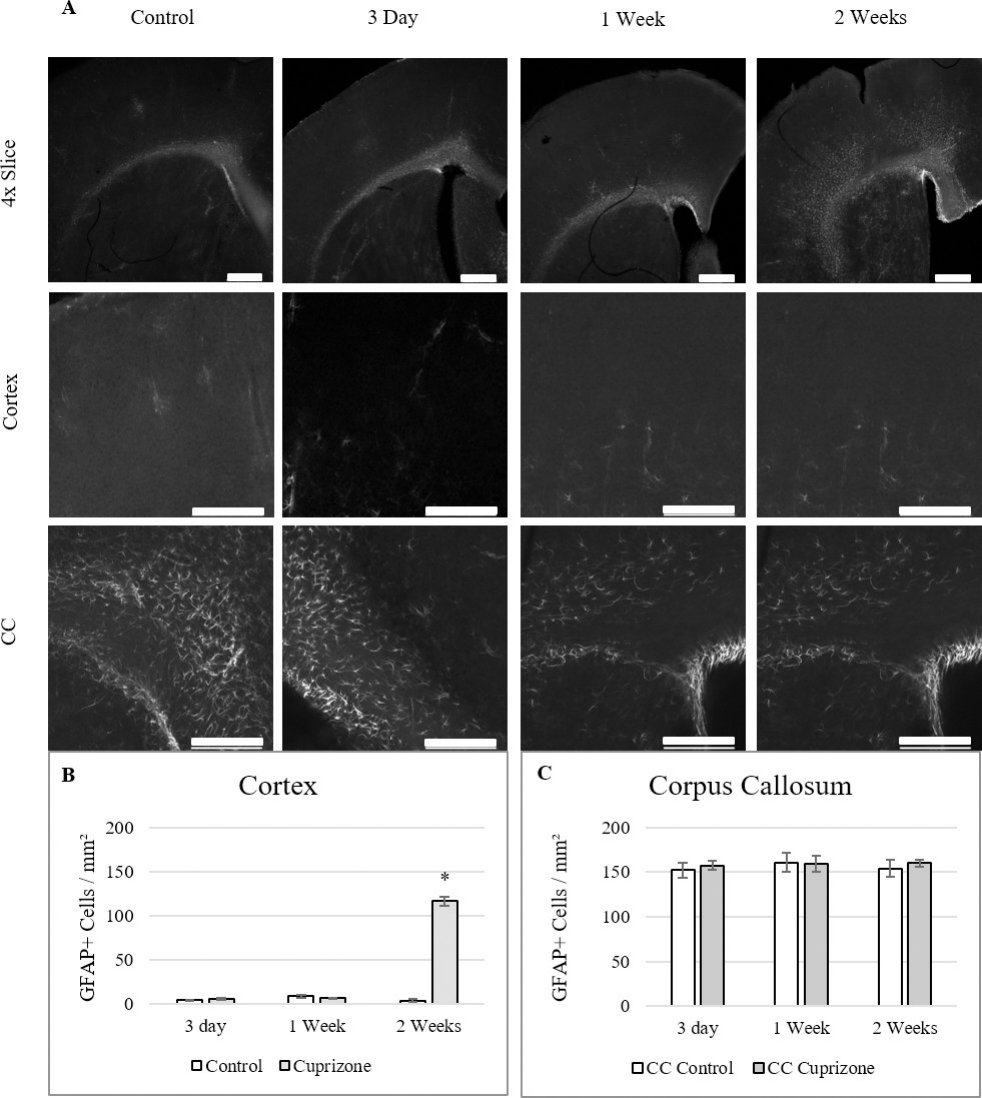
GFAP^+^ Astrogliosis occurs after 2 weeks of cuprizone administration. (A) 4x images show changes across cortical and corpus callosum. Cell counts were taken from 20x Cortex and 20x Corpus Callosum regions of each slice. (B-C) Quantification of GFAP^+^ astrocytes within both regions (mean ± s.e.m.). Reactive gliosis was significantly increased by 2 weeks in cortical tissue but not in the corpus callosum over the control tissue. 4x Scale bar = 500 μm, 20x Scale bar = 200 μm, *n* = 6. Asterisk denotes significance (*p* = < 0.05).

To better understand how the BBB is altered during the early stages of cuprizone administration, the timing of gliosis of astrocytes and microglia was examined through the first 2 weeks of cuprizone. Both astrocytes and microglia have been shown to alter BBB integrity after immune activation,[44,45] so their activation and growth was measured during the first 2 weeks to determine if they may contribute to BBB hyperpermeability. Astrocyte activation was measured using GFAP fluorescent staining in cortical and corpus callosum tissue **(Fig 3A)**. Image thresholding was performed on 20x images and astrocyte cell counts were performed on the thresholded cells to reduce background and prevent re-counting. Cuprizone treatment induced significant activation of GFAP^+^ astrocytes in cortical tissue at 2 weeks (116±13) over control (4±3) **(Fig 3B).** Within the corpus callosum, higher baseline levels of GFAP^+^ astrocytes existed in both the control and cuprizone treated mice, showing no significant increase in the 2 weeks of treatment **(Fig 3C)**.

**Fig 4:**
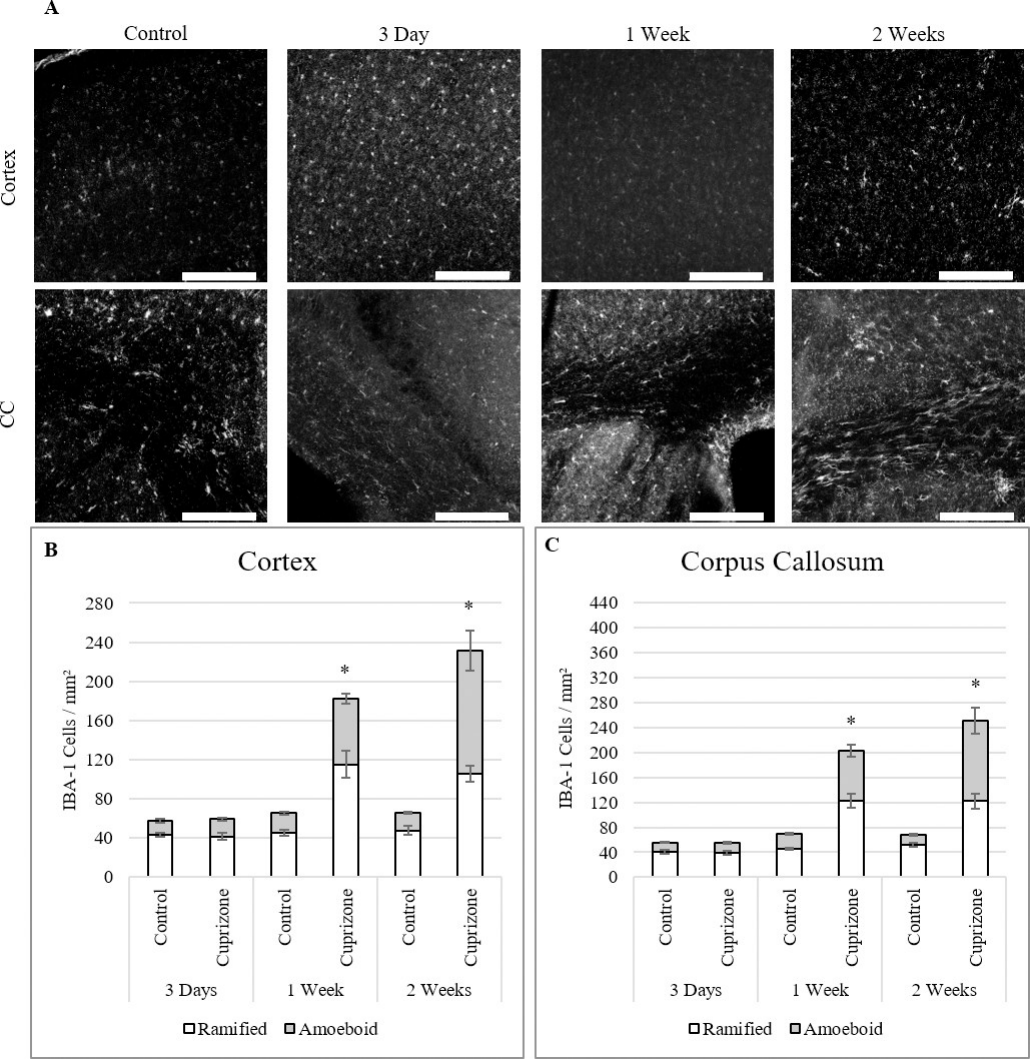
Gliosis of IBA-1 stained microglia through 2 weeks of cuprizone administration. (A) Fluorescent images of IBA-1 stained microglia in both cortical and corpus callosum regions. (B-C) Total counts of microglia increased significantly in both cortex and corpus callosum after 1 week of cuprizone administration. Within the total counts, there was significant increases at 1 week for both inactive and active morphologies of microglia in both regions (mean ± s.e.m.). 20x Scale bar = 200 μm, *n* = 6. Asterisk denotes significance (*p* = < 0.05).

Microgliosis and microglial activation was measured via IBA-1 antibodies in cortical and corpus callosum tissue **(Fig 4A)**. 20x images were run through a threshold for cell counts and morphology was determined through blinded count. Within both the cortex and corpus callosum, significant total microglial count was measured at 1 week in cuprizone treated mice (115±13 cortex, 122±11 CC) over controls (45±7 cortex, 45±2 CC) **(Fig 4B and 4C)**. Within the total count, both the unactivated, ramified and the activated, amoeboid morphologies increased significantly at 1 week for both the cortex and corpus callosum. This puts microglial activation before astrogliosis, but it still occurs after the BBB alterations observed were at their greatest, implicating another source for the cause of the hyperpermeability.

### Mast cell activation coincides with peak BBB hyperpermeability

**Fig 5:**
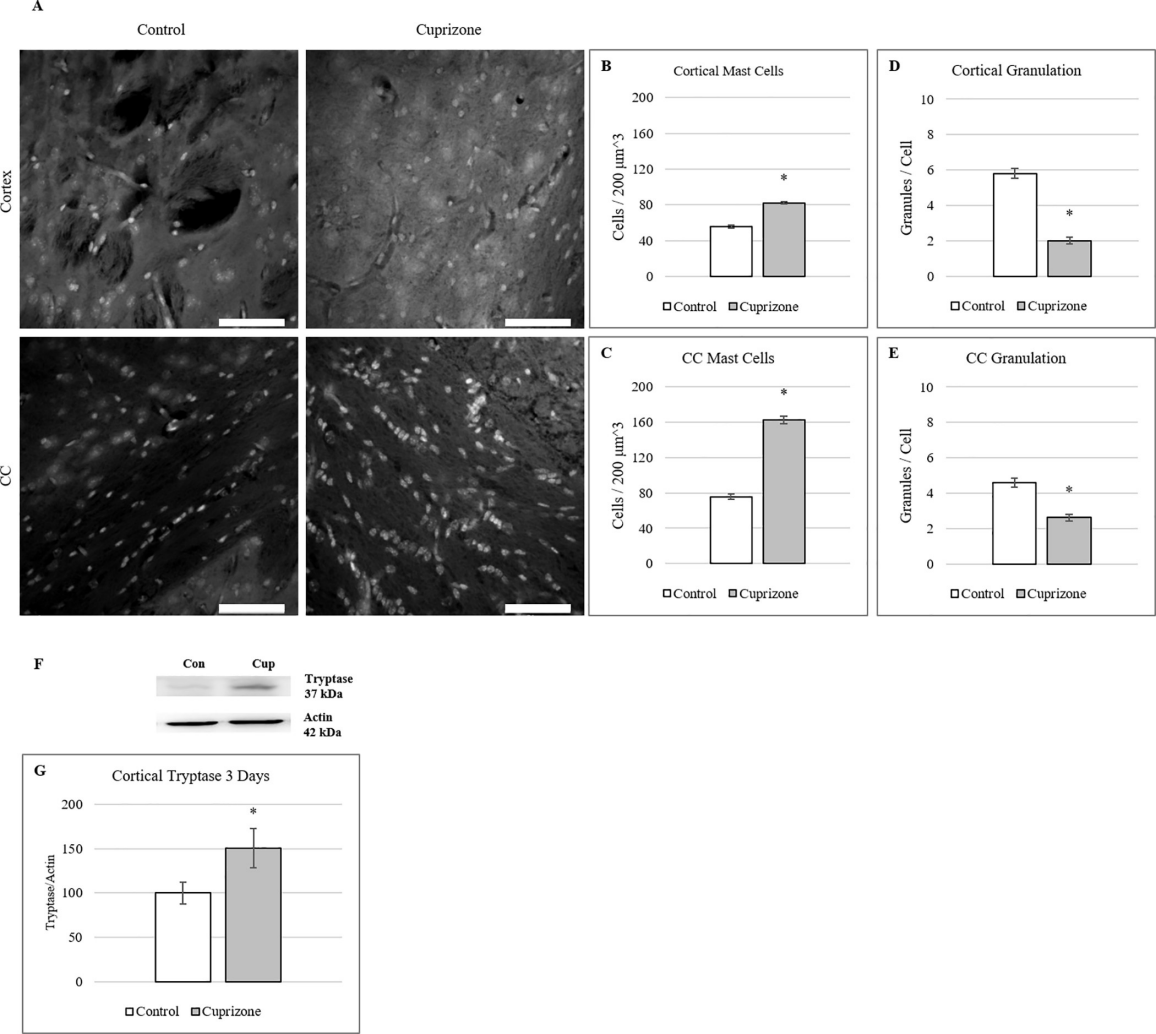
Mast cell activation occurs at 3 days of cuprizone administration. (A) Toluidine Blue stained mast cells in cortical and corpus callosum tissue after 3 days of cuprizone administration. (B-C) Quantification of mast cell presence shows significant increases in both cortical and corpus callosum tissue after 3 days of cuprizone administration. (mean ± s.e.m.). *n* = 6 (D-E) Measures of mast cell degranulation shows a decrease in stained mast cell granules in both cortical and corpus callosum tissue after 3 days of cuprizone administration. *n* = 30 (mean ± s.e.m.). Scale bar = 50 μm, Asterisk denotes significance (*p* = < 0.05) F) Western blot analysis of cortical Tryptase at 3 days of cuprizone administration. Representative blots of Tryptase and Actin in mice after 3 days of cuprizone administration. G) Densitometry of western blot shows significant increase in Tryptase levels compared to controls. Data are expressed as a percentage of control with the highest control set at 100%. *n* = 3. Asterisk denotes significance (*p* = < 0.05).

Having observed hyperpermeability from the vasculature in the brain as early as 3 days into cuprizone administration, mast cells, known for their rapid activation and response to insult [46] were examined at 3 days of cuprizone treatment to determine if their activity was increased during this timepoint. Mast cell presence in cortical and corpus callosum tissue[47–49] was measured via fluorescent imaging of toluidine blue staining **(Fig 5A)**.[36] Cell and granule counts were performed on thresholded images and counted by a blind observer. Mast cells showed significantly higher populations in both cortical (82±1 cup vs 56±2 con) **(Fig 5B)** and corpus callosum (87±12 cup vs 75±3) **(Fig 5C)** tissue at 3 days of cuprizone administration. Degranulation of mast cells was also counted in stained tissue under fluorescence. Both cortical **(Fig 5D)** and corpus callosum **(Fig 5E)** mast cells showed significant decrease in granule staining at 3 days compared to controls at 3 days indicating release of granule products such as histamine which has been shown to increase BBB hyperpermeability.[50]

Tryptase, a known constituent of mast cell granules and an indicator of mast cell activity,[51] was examined via western blot at 3 days of cuprizone (**Fig 5F)**. Protein levels were significantly increased by 1.5-fold at 3 days in cortical tissue but although corpus callosum levels were higher in cuprizone treated mice, no significant increase was measured **(Fig 5G)**. These increased levels indicate that mast cells were increased in response to cuprizone treatment and that their increased activity coincided with the highest levels of BBB permeability.

## Discussion

The cuprizone model serves as a valuable tool for the investigation of toxically induced changes within the CNS. Much of the current studies focus on the end effects of cuprizone administration on the CNS and how they may be prevented or repaired. However, it is still not well understood exactly how cuprizone creates the demyelination observed which limits the ability to precisely study and rectify the consequences of cuprizone administration. Mice were fed a diet of 0.3% cuprizone to provide the maximum tolerable dose of cuprizone without adverse liver toxicity.[33] Both corpus callosum and cortical regions, known to be affected by cuprizone, were examined. Observing weight-loss patterns both observed and reported[33,52–54], timepoints of 3 days, 1 week, and 2 weeks were established to investigate the early effects of cuprizone. The aim of the current study was to better understand the early effects of cuprizone administration on the CNS and how the BBB is affected.

Historically, it has been shown that cuprizone administration does not alter BBB integrity.[23,26] However emerging evidence[24] has suggested that BBB alterations do occur under cuprizone, and that they occur very early on during cuprizone administration. It was suggested that the increase in permeability found as early as 5 days was likely due to released astrocytic factors such as TNF-α, Ccl2, Il1-β, and Il6. The release of these factors occurs before measurable, morphological activation of GFAP^+^ astrocytes as shown in this study, showing that finer timepoints during the beginning of cuprizone administration need to be examined. This study has provided further evidence of BBB hyperpermeability and that it occurs as early as 3 days after cuprizone treatment. This timing suggests that potentially, the breakdown of BBB integrity may be an important event in the cascade of activation seen in cuprizone. In certain insults such as ischemia, alterations in the BBB can occur as early as 2 hours after reperfusion and that this breakdown is essential for the progression of reperfusion injury seen during ischemic events.[55,56] This shows that BBB alterations may occur quickly and that examinations of BBB permeability within the cuprizone model need to be performed at finer timepoints from the outset of cuprizone administration to better understand when the BBB initially begins to disintegrate and what chemical signals are present which may contribute to the BBB alterations. The significant decrease in the tight junctional protein Occludin at 3 days within the corpus callosum measured in this study shows that as early as 3 days, changes in protein expression are occurring at the BBB. This finding is in line with *in vitro* evidence of decreased occludin expression in endothelial cultures treated with cuprizone for 48 hours.[24] This helps to explain the observed hyperpermeability as tight junction proteins such as occludin are responsible for maintaining the integrity of the barrier by cementing adjacent endothelial cells tightly together.

The increase in mast cell presence in both the cortex and corpus callosum as well as cortical increases in tryptase expression shows activation of this cell population during cuprizone administration, a novel finding in the examination of cuprizone-related BBB alterations. Mast cells are known to be early effectors of immune response within tissues [29] and are often located in the perivascular space inside the BBB, closely associated with blood vessels.[57,58] They have been shown to release factors known to induce BBB permeability changes.[32] Mast cell presence was determined using the fluorescence of toluidine blue to image their presence in thick tissue sections. A significantly higher presence of mast cells in both cortex and corpus callosum was measured as well as significantly decreased granulation of the cells in both regions. This finding agrees with the measure of increased mast cell tryptase in the cortex at 3 days. Tryptase is commonly used as a measure of mast cell degranulation [59] and the increase of tryptase in the cortex coincides with the increase in BBB permeability seen at 3 days. These findings suggest that mast cells may play a pivotal role in the initiation of the effects seen in the CNS under cuprizone administration. Hirase et al. showed that release of granule contents such as histamine and lysophosphatidic acid (LPA) from mast cell granules has been shown to increase paracellular permeability of horseradish peroxidase in endothelial cell cultures.[60] They found that this increase coincided with phosphorylation of occludin within the endothelial cultures. Elevated phosphorylation of tyrosine residues on occludin have also been linked to tight junctional disruption.[61] These findings link mast cell activation to BBB degeneration through occludin disruption. This mirrors the findings of this study implicating the release of granule contents from activated mast cells in the decrease in occludin expression and subsequent increase in BBB permeability. Mast cells are also known to be sources of chemokines such as CCL2 and cytokines such as TNF-α that are known to alter BBB integrity and activate glial populations.[62–64] However, further analysis of mast cell participation is necessary to better understand how they are affected by cuprizone administration. Mast cell stabilizers such as cromoglycate or more targeted inhibitors such as antihistamines like famotidine or cimetidine would aid in evaluating the level of mast cell involvement in BBB alterations and by what pathways these changes occur.

When mast cells are activated, they have been observed to release granule contents within minutes and up-regulate the synthesis of many granule components within hours.[63] Mast cells are activated most commonly by IgE bound to high affinity receptors (FcεRI) or via c-Kit/stem cell factor receptor pathways.[65] However, mast cells have also been shown to activate production of IL-4 and IL-6 and induce degranulation in the presence of reactive oxygen species (ROS) via an APE/Ref-1 dependent pathway.[66] This pathway could explain mast cell activation during cuprizone administration, and the subsequent BBB degeneration shown to occur. The mitochondrial changes seen in oligodendrocytes caused by cuprizone administration increases ROS and increased oxidative stress which leads to apoptosis.[5,67,68] The presence of these ROS and apoptotic cells may activate the mast cells to degranulate and aid in the alterations of the BBB seen. Mast cells may also aid in the activation of microglia observed within 1 week of cuprizone treatment via the release of tryptase due to this activation.[69] It is important to in future to evaluate the specific pathway in which mast cells become activated Further examinations into these pathways will be important in understanding how mast cells specifically react to cuprizone treatment.

The findings of this study implicate mast cells as potential sources of BBB disruption during cuprizone treatment. However, further examination of this population needs to be performed. Mast cells are known Understanding the mode of activation for these cells, as well as the specific chemicals they release during this activation may lead to a better understanding of how the BBB is disrupted during cuprizone. Antihistamines or other mast cell product antagonists might be utilized to prevent the breakdown of the BBB at the early timepoints within cuprizone treatment. If so, the effects of an intact BBB on pathological progression or lack thereof should be investigated to better determine the role of BBB degeneration in cuprizone pathology. This could allow for better targeted treatments for diseases such as MS in which both BBB degeneration and mast cell activity are a feature.[70,71] They are present around demyelinated lesions[72], suspected to be involved not only in BBB disruptions, but also in the regulation of the autoimmune response and to aid in demyelination.[70,73] These findings have also been found in the animal model experimental autoimmune encephalomyelitis (EAE) that has a larger autoimmune component than the cuprizone model does.[74]
